# ^1^H-NMR-Based Metabolomic Profiles of Zucchini (*Cucurbita pepo* L.) Grown with Different Agricultural Practices for Sustainable Crop Production

**DOI:** 10.3390/foods14060919

**Published:** 2025-03-07

**Authors:** Miriana Carla Fazzi, Chiara Roberta Girelli, Danilo Migoni, Beatrice Fracasso, Gianluigi Cesari, Francesco Paolo Fanizzi

**Affiliations:** 1 Department of Biological and Environmental Sciences and Technology, University of Salento, 73100 Lecce, Italy; mirianacarla.fazzi@unisalento.it (M.C.F.); chiara.girelli@unisalento.it (C.R.G.); danilo.migoni@unisalento.it (D.M.); beatrice.fracasso@unisalento.it (B.F.); 2 Centre International de Hautes Etudes Agronomiques Méditerranéennes, Mediterranean Agronomic Institute of Bari, Via Ceglie 9, 70010 Valenzano, Italy; cesari@iamb.it

**Keywords:** zucchini, ^1^H-NMR spectroscopy, metabolomics, agricultural practices, sustainability, food products, nutraceutical properties

## Abstract

Zucchini (*Cucurbita pepo* subsp. *pepo*) is a seasonal vegetable (also known as courgette) characterized by health properties due to the content of several bioactive molecules. For this reason, the consumption of zucchini is highly recommended as a part of the Mediterranean diet. The aim of this study was to evaluate the possible influence of a specific compost supply for shifting the characteristics of an integrated agriculture toward a biodynamic standard following Demeter^®^ certified rules. In particular, an approach based on ^1^H-Nuclear Magnetic Resonance (NMR) spectroscopy and multivariate statistical analysis (MVA) was applied to analyze the differences between the metabolic profiles of the zucchini samples (with the same cultivar, *Vitulia*), obtained from three different agronomical practices: two focused agricultural systems (compost supplied and integrated), as well as the used benchmark (Demeter biodynamic certified). The obtained results showed that the samples from the plots managed with biofertilizer from compost showed similar behaviour to the samples managed under Demeter biodynamic certification, with higher content of some amino acids, such as arginine, and lower content of sugars than the samples from integrated farming. The concentration of twenty elements was then determined using inductively coupled plasma atomic emission spectroscopy (ICP-AES). The averaged results of the elemental data appear almost parallel to the trend observed with the metabolomics approach. In the present case, the use of a specific compost as a biofertilizer has shown to promote the transition to the quality standards of the Demeter certification, significantly improving the crops’ sustainability.

## 1. Introduction

Zucchini (*Cucurbita pepo* subsp. *pepo*) is a seasonal vegetable (courgette) characterized by health properties due to its content of several bioactive molecules such as phenolic compounds and organic acids [1]. Specifically, the nutraceutical and health potential of zucchini is due to the presence not only of these components, but also of water-soluble and liposoluble vitamins, and mineral salts, specifically potassium [2,3]. For this reason, the consumption of zucchini and other seasonal vegetables is highly recommended as part of the diet, particularly the Mediterranean diet [4,5]. The composition of vegetables, as important components of the Mediterranean diet, determines not only the quality and safety of the product, but also its nutritional properties [6]. Moreover, the Mediterranean diet is considered a sustainable diet due to its significant nutritional and health benefits, minimal environmental effects and abundance of biodiversity, strong socio-cultural food values, and local economic advantages [7].

Therefore, it is important to evaluate the composition of foods, and specifically of vegetables, because they are an essential part of the sustainable Mediterranean diet. In addition, the consumption of “natural” food products has been increasingly encouraged since the negative effect of agriculture on the environment has increased with the intensification of food production [8]. On the other hand, the characteristics of zucchini and other vegetables are influenced not only by climatic and environmental conditions [9] but also by cultivation methods [10]. This encourages the application of more sustainable and eco-friendly agricultural practices, which limit or avoid the use of synthetic chemicals [11].

Among the most sustainable agricultural practices, the application of the biodynamic method [12] and the use of compost as a fertilizer [13] are considered in contrast with integrated agriculture. Specifically, biodynamic agriculture is described as “an unintegrated agriculture method of agriculture” (Steiner, 1924) and is currently considered “one of the approaches of organic agriculture” [14,15]. This method was established by the international certification mark Demeter^®^ [16]. It is based on a closed production system that aims to replicate an agroecological model focused on reducing energy consumption and enabling high levels of efficiency [15] and positive environmental impact [12]. Biodynamic agriculture is kind of organic agriculture because they share some principles, but Demeter’s production rules further provide for restrictions on many organic farming practices [15]. The International Federation of Movements for Organic Farming (IFOAM) defined the main differences between the Demeter^®^ standards and the organic production standards [17]. Demeter^®^ rules also prescribe the use of specific biodynamic preparations (Table S1) [11,18]. In the present work, the general definition of biodynamics specifically refers to the organic certified Reg. CE 848/18 [19] Demeter^®^-compliant [16] production rules. Meanwhile, composting can be defined as the “process whereby microorganisms convert organic material into a biostable product” [20]. The increasing use of compost in agriculture necessitates an assessment of its environmental benefits and impacts compared to other crop improvers and soil fertilizers [21]. The quality of composts made from different organic wastes depends on the composition of the raw material used [22]. Therefore, compost supply process has many advantages such as the recovery of organic waste and the improvement of the chemical–physical and biological properties of the soil, with environmental, agronomic, and economic benefits [23]. In this study, the effect of a specific compost supply (Bio Vegetal) was evaluated. Bio Vegetal is a biofertilizer enriched with humus and live microorganisms; it was designed to restore the natural equilibrium of the soil, improving the vegetative state of the plants and increasing the productivity of the crop (Table S2) [24]. This compost is produced by the Apulian farm Tersan, using an industrial composting method under controlled conditions, which are characterized by greater speeds than the natural process [25].

On the other hand, industrialised production has some advantages, such as yield maximisation, but it involves the use of chemical substances [26]. Thus, although the industrial model can achieve high yields and profits in the short term, they depend on high long-term costs [27] and are associated with losses related to soil fertility, biodiversity, and the nutritional quality of crops [28]. Indeed, integrated agriculture, or the industrial model, involves using a considerable amount of chemical fertilizers and pesticides to increase production [29]. For this reason, both in public debate and in the scientific literature, disputes about the future are increasingly focused on the comparison between “alternative” and “integrated agriculture” [30]. In this respect, biodynamic agriculture and composting are sustainable methods for food production as alternative approaches.

Therefore, the aim of this study was to evaluate the effects of different agricultural practices on zucchini’s metabolic profiles and related nutritional properties by using an untargeted ^1^H-NMR-based metabolomics approach [11,31,32]. The untargeted approach was here carried out via the ^1^H-NMR-data-based metabolic fingerprinting of the samples for comparative purposes. The subsequent profiling of the significant discriminant assigned metabolites with relative quantification was then calculated as fold change ratios [33,34]. Metabolomics is a scientific method used in food sciences for evaluating the authenticity and traceability of agri-food products [35,36]. Among the analytical techniques used in metabolomics, NMR is one of most widely applied for food science and nutrition research because it is a non-invasive, powerful, and dependable quantitative method that offers an examination of each molecular element within an elaborate matrix [11,32,37,38,39,40,41].

The ^1^H-NMR metabolomic approach has already been used to study the variations in zucchini’s metabolic profiles, focusing on the possible effects of different water irrigation systems [31] and storage conditions (with the zucchini packed in both compostable and plastic containers) [3]. Using the same metabolomic approach, Picone et al. and Gallo et al. studied the possible effects of biodynamic vs. organic [42], and organic vs. conventional [43] agricultural methods, respectively, on table grapes. More recently, the metabolomics approach was applied to investigating the effects of biodynamic, organic, and integrated agriculture on table grape juice [11]. Furthermore, the metabolic profiles of other agri-food matrices produced using different agricultural practices have been investigated. Thus, the metabolic profiles of *Brassica oleracea* [44] and early potatoes [45], grown using organic and conventional methods, were investigated. To the best of our knowledge, this is the first study selectively focused on the possible influence of a specific compost supply [24] in terms of shifting the characteristics of integrated agriculture toward a biodynamic that refers to the organic certified Reg. CE 848/18 [19] Demeter^®^-compliant [16] production rules.

## 2. Materials and Methods

### 2.1. Zucchini Samples’ Origin, Collection, and Preparation

**Figure 1:**
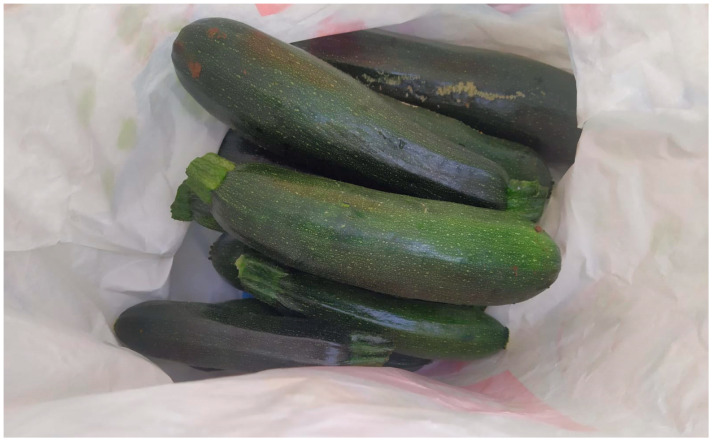
Zucchini of the cultivar *Vitulia* (Syngenta CV 2832) described in this study.

A specific investigation was carried out into zucchini juices samples from the same cultivar *Vitulia* (Syngenta CV 2832), (Figure 1 and Figure S1), obtained using the three investigated cultivation methods (biodynamic, compost supplied, and integrated agriculture). Three replicates were obtained from each of the five selected zucchini samples, representative of the cultivation methods (15 samples/method), for a total of 45 samples.

The plant material was obtained from the same nursery, which is certified in organic farming (Azienda Agricola Junior Plant Fasano, Brindisi, Italy), and it was then transplanted in the two collaborating specialised Italian farms, located in the south of Italy (Apulian Region). Lacalamita Rosa (Castellaneta, Taranto, Italy) provided the biodynamic farming while Tenuta Pinto (Mola di Bari, Bari, Italy) was involved in the compost-supplied and integrated agriculture trials. Their geographical coordinates are, respectively, 40°34′45″ N 16°56′26″ E altitude 70 m (biodynamic farm) and 41°01′45″ N 17°02′57″ E altitude 117 m (biofertilizer from compost-supplied and integrated agriculture management) (Figure S2). In the present investigation, biodynamics refers to the organic certified farm Lacalamita Rosa (Castellaneta, Taranto, Italy) Reg. CE 848/18 [19] following Demeter production rules (Biodynamic Federation Demeter International) [16]. The used soils were managed according to the Demeter standard protocol in operation since 2005.

At the Lacalamita Rosa farm in the countryside of Castellaneta (TA) in Contrada Salesiani, seedlings were transplanted in the first ten days of May. At the Tenuta Pinto farm, the plots were defined and fertilization was prepared in April 2023 for the compost-supplied experiments; meanwhile, in the other plots (integrated agriculture), pre-transplant fertilization was carried out according to ordinary farming practices. The transplanting of nursery seedlings was also performed in the first ten days of May for the Tenuta Pinto farm experiments.

Sample collection occurred at the two farms on 21 June 2023. On the same day, samples, provided by the Apulian farmers with their informed consent, were delivered to the Department of Biological and Environmental Sciences and Technology laboratories (University of Salento) and stored at −20 °C until further processing.

### 2.2. NMR Chemicals and Reagents

All chemical reagents for analysis were of analytical grade. Deuterium oxide (99.9 atom %D) containing 0.05% wt 3-(trimethylsilyl)propionic-2,2,3,3 d4 acid sodium salt (TSP) potassium phosphate monobasic was purchased from Armar Chemicals (Döttingen, Switzerland). Sodium azide was purchased from J.T. Baker (Phillipsburg, NJ., USA).

### 2.3. Sample Preparation and NMR Spectroscopy

Juice was obtained from the zucchini samples for each different cultivation by pressing 10–15 g of flesh with peel. Each juice sample was prepared in three replicates obtained from each of the five zucchinis that were representative of a specific cultivation method; analytical reproducibility was evaluated by overlapping technical replicates in MVA (PCA and PLS-DA) score plots [32]. For ^1^H-NMR direct analysis of the zucchini juices, 100 μL of 90 mM KH_2_PO_4_ buffer in D_2_O (pH 6), containing 0.05% *w*/*v* TSP-d4 (sodium salt of trimethylsilylpropionic acid) as an internal standard and Sodium azide to prevent microbial contamination [46], were added to 900 μL of juice and subsequently centrifuged at 10,000 rpm for 5 min at room temperature (23 °C). Then, 600 μL of the supernatant was placed into a 5 mm NMR tube. A Bruker Avance III 600 Ascend NMR spectrometer (Bruker Italia, Milano, Italy), operating at 600.13 MHz and equipped with a BBO probe with a z-axis gradient coil and automatic tuning-matching (ATM), was used to perform all measurements. Experiments were run at 300 K in automation mode after individual samples were loaded on a Bruker Automatic Sample Changer, interfaced with the software IconNMR version 5 (Bruker) software. For each sample, the acquisition of the ^1^H-NMR spectra was executed with pulseprogram zgcppr (Bruker) to suppress the water signal, in a spectral window of 20.0276 ppm (12,019.230 Hz), 64 scans and a 90° pulse of 10.160 µsec. After acquisition, standard processing procedures were performed on the acquired free induction decay (FID), using TopSpin 3.6.1 software (Bruker, Biospin, Italy). These included the application of an apodization function with a line broadening of 0.3 Hz and the Fourier transform (a mathematical operation that converts the free induction decay into a spectrum of frequencies), followed by phase and baseline correction. All NMR spectra were calibrated using the internal standard signal TSP (δ = 0.00 ppm). The metabolites were identified and assigned based on 2D NMR spectral analysis (2D ^1^H Jres, ^1^H COSY, ^1^H-^13^C HSQC and HMBC). The assignments were confirmed by a comparison with data from the literature [31,32]. The acquisition and processing of the 2D spectra were performed as follows:

First, 2D homonuclear shift correlation (cosygpprqf Bruker standard pulse program), which was carried out with presaturation during relaxation delay. This was obtained with spectral width in both dimensions 12,019 Hz (20.028 ppm), 8 scans, 16 dummy scans, 2048 data points in f2, 512 increments in f1; data were processed with an unshifted sine-bell squared (QSINE) window function in both dimensions before the Fourier transform.

Then, the ^1^H homonuclear J-resolved spectrum (Bruker jresqf pulse program). We used a spectral width of 12,019 Hz for the f2 dimension (chemical shifts axis) and 80 Hz for f1 (spin–spin coupling constant axis), 16 scans, 16 dummy scans, 2048 data points in f2, and 256 increments in f1. There was a 2 s relaxation delay with a total acquisition time of 4 h and 11 min. The spectrum was processed with zero filling in f1 to 4096 real data points, with unshifted sine-bell squared window (QSINE) functions in both dimensions before the Fourier transform.

Then, the ^1^H-^13^C gradient-selected HSQC spectrum (Bruker hsqcetgpsisp2.3 pulse program) was acquired via ^1^H-^13^C decoupling, 9 and 45 kHz spectral widths in the ^1^H and ^13^C dimensions, respectively, 8 scans, 16 dummy scans, 2048 data points in f2, 1024 increments in f1, forward linear prediction with 32 coefficients, and zero filling to 4096 data points for the f1 dimension. Unshifted sine-bell squared (SINE) window functions were also applied in both dimensions before the Fourier transform.

The ^1^H-^13^C HMBC spectrum was obtained with 9 and 45 kHz spectral widths in the ^1^H and ^13^C dimensions, respectively, 4 scans, 4 dummy scans, 2048 data points in f2, 512 increments in f1, forward linear prediction with 32 coefficients, and zero filling to 4096 data points in f1. Unshifted sine-bell squared (SINE) window functions were also applied in both dimensions before the Fourier transform.

The resulting 2D spectra are reported as Figures S3–S6.

### 2.4. NMR Data Processing and Statistical Analysis

After the spectral data processing step, ^1^H-NMR spectra were segmented in rectangular buckets (0.04 ppm width) and integrated using the Bruker Amix 3.9.14 (Analysis of Mixture, Bruker BioSpin GmbH, Rheinstetten, Germany) software to transform the NMR spectra into a format suitable for multivariate analysis. The process involving the segmentation of each NMR spectrum into regions or histograms with a fixed base width of 0.04 ppm is also known as “normal rectangular bucketing”. The residual water spectral region was excluded from the buckets; the reduced spectra and the remaining 233 buckets in the range 10.00–0.50 ppm were then normalized to the total intensity (to reduce small differences between samples due to the metabolite concentration and/or experimental conditions) and subsequently mean centred to reduce the variability and noise in the data. The Pareto scaling method was then applied (for the optimal accounting of small and large variables), which involved dividing each variable by the square root of the variable standard deviation centred around the mean value [47,48].

Therefore, the data table, obtained with all the standard aligned and bucket-reduced NMR spectra and composed of rows (observations/samples) and columns (variables/buckets), was used in the multivariate data analysis. Each bucket, in a bucket row of representatives of the spectrum, was labelled with the central chemical shift value for its specific 0.04 ppm width. The integral values of each row of buckets represent the variability of each sample that can be used for statistical analysis. After data processing, MVA was performed using Simca-P version 14 (Sartorius Stedim Biotech, Umeå, Sweden) software. Specifically, unsupervised (principal component analysis, PCA) and supervised (orthogonal partial least squares discriminant analysis, OPLS-DA) pattern recognition methods were used to examine the intrinsic data variation. From the data table, PCA can extract and show systematic variation in an X-data matrix without using group identity information to build the model. Indeed, PCA was the first analysis performed because it is the basis of multivariate analysis [49] and provides an overview of all observations in the data table [48]. Moreover, PCA is generally used to obtain a general description of the samples’ distribution and their possible grouping in homogeneous clusters [50]. PCA can also be used to detect groupings, trends, and outliers [51]. After observation of the PCA, to further maximise the separation between classes of samples, OPLS-DA was applied. OPLS-DA is a potent technique for the interpretation and classification of sample sets. This supervised analysis is an extension of PLS-DA (partial least squares discriminant analysis) modelling [52]. PLS-DA is the regression extension of the PCA, which gives the maximum covariance between the measured data (variable X, metabolite matrix bucket in NMR spectra) and the response variable (variable Y, class matrix) [53]. Therefore, they are used to discriminate samples grouped into classes with different characteristics. OPLS-DA can detect the most discriminating variables, filtering the variation that is not directly related to the focused discriminant response. The part of the variance useful for predictive comparisons is separated from the non-predictive part, which is then considered orthogonal. As a result, the interpretability of the model is greatly improved [54]. The statistical models were validated using the internal cross-validation default method (7-fold) and further evaluated with permutation tests (100 permutations), which are all available on the SIMCA-P software version 14 [48]. The quality of the models was described using the R^2^, Q^2^, and p[CV-ANOVA] parameters. R^2^ is a cross-validation parameter defined as the part of the variance in the data explained by models; it indicates the goodness of fit. Q^2^ represents the portion of variance in the data predictable by the model. It is a measure of the predictive ability of the model, describing how well it can predict correct classifications for a test sample. The minimal number of components required can be easily defined since the R^2^(cum) and Q^2^(cum) parameters demonstrate completely differing behaviours as the model complexity increases [55]. In the supervised analysis, the cross-validated variance analysis (CV-ANOVA) provides a *p*-value that describes the level of significance of the separation of groups [54,55]. Moreover, permutation tests and CV-ANOVA were used to validate the predictive capacity and statistical significance of the OPLS-DA models. Additionally, the *p*-values of CV-ANOVA below 0.05, and all permuted R^2^ and Q^2^ values with the intercepts of R^2^ and Q^2^ values on the Y-axis being <0.5 and <0, respectively, indicate a good fit for the OPLS-DA models [48]. Finally, the model’s predictive ability was confirmed using a misclassification table with Fisher’s exact test [56].

In order to find the discriminating metabolites responsible for the separation between the classes of samples, the tools available on the SIMCA-P software were used. For the PCA model, a loading scatter plot was used to show the contribution of the original variables that describe each sample (buckets obtained from NMR spectra) and define the samples’ position in the score scatter plot. Another tool often used for the OPLS-DA model with two classes is the S-line plot. This method visualizes the centred loading vector p(ctr), coloured according to the absolute value of the correlation loading p(corr) and distributed as positive and negative values (with reference to the two compared classes) in a form similar to the original spectra. The relative changes in the metabolite content between the two groups of samples was quantified as the ratio of the standardized median intensity of the corresponding selected buckets (representative of specific NMR undistorted signals for each discriminating metabolite) in the pairwise comparisons (fold change) [37,57]. The statistical significance of the differences for each variable of the two groups in the pairwise comparison was evaluated using the Student *t*-test. In order to classify a difference as statistically significant, in this work, only metabolites with *p*-values < 0.05 (confidence level 95%) were considered [11,32]. Moreover, only metabolites with a VIP (variable importance in projection), estimated from all extracted components, and a correlation coefficient p(corr), respectively, with absolute values higher than 1.0 and 0.5 were considered as possible significant discriminants and included in the relative quantification [58].

### 2.5. Measurement of Element Content via ICP-AES Analysis

A total of twenty elements were analysed in the zucchini samples: calcium (Ca), phosphorus (P), cadmium (Cd), copper (Cu), manganese (Mn), iron (Fe), chromium (Cr), cobalt (Co), nickel (Ni), lead (Pb), potassium (K), magnesium (Mg), barium (Ba), strontium (Sr), beryllium (Be), lithium (Li), tellurium (Te), thallium (Tl), arsenic (As), and zinc (Zn). The concentrations were determined using inductively coupled plasma atomic emission Spectroscopy (ICP-AES), by following the standard procedures. Three replicates were obtained from each of the five selected zucchini samples that were representative of the cultivation methods (15 samples/method) for a total of 45 samples. The zucchini were washed with water and wiped to remove all the soil particles. For each sample, 0.5 g of fresh weight was mixed with 4 mL H_2_O_2_ and 6 mL HNO_3_ at 180 °C for 10 min, using a microwave digestion system (Milestone START D) [59]. The sample was then cooled, diluted with ultrapure water to a final volume of 20 mL, and filtered through Whatman No. 42 filter papers, and the final solutions were subsequently analysed using a Thermo Scientific iCAP 7000 ICP-AES spectrometer to determine the elemental content. The element concentrations were expressed as mg/kg dry weight (ppm) and results are presented as the mean (±standard deviation) for the different measurements obtained for each of the three cultivation methods [60]. Mean values were compared using ANOVA followed by Tukey’s HSD test at (*p* < 0.05) [59] using the MetaboAnalyst 6.0 software [61].

## 3. Results and Discussion

### 3.1. ^1^H-NMR Spectrum of Zucchini Juice Sample and Assignment of the Peaks

**Figure 2:**
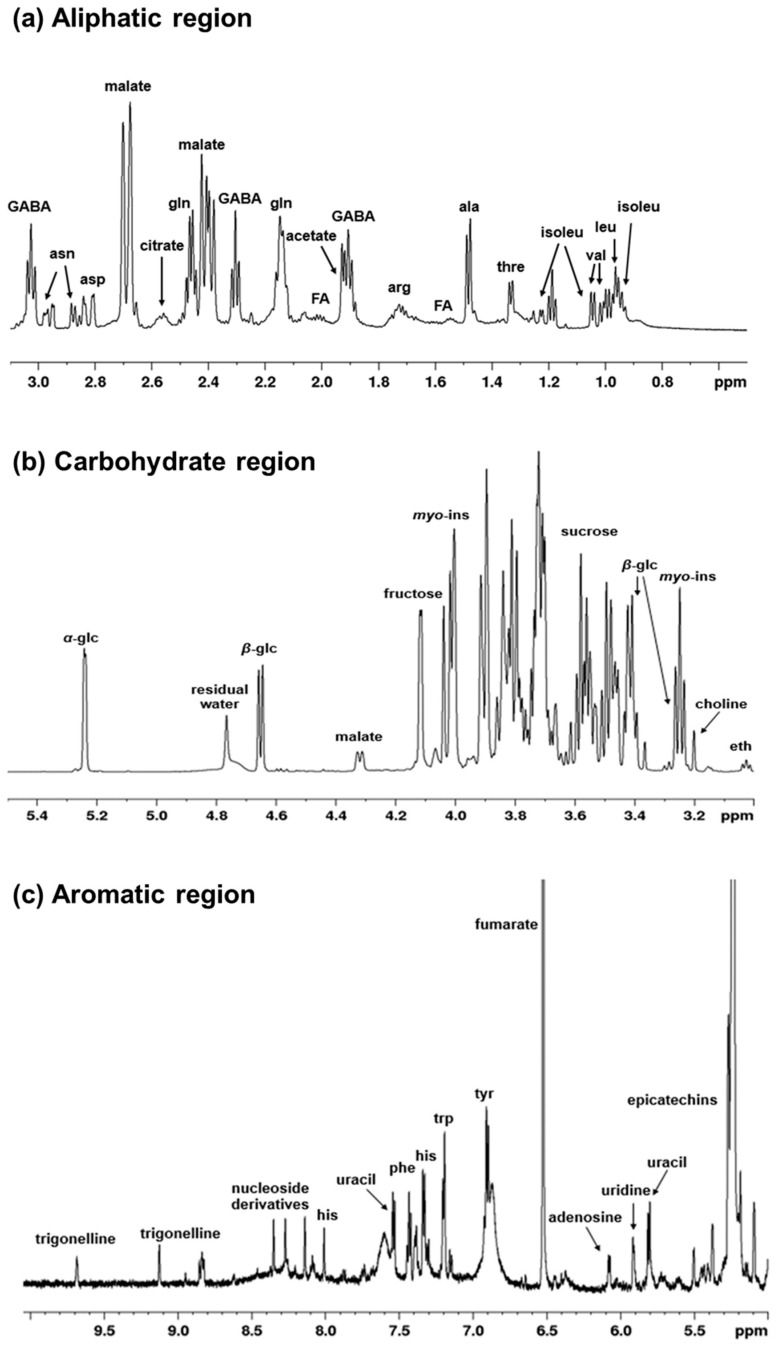
^1^H-NMR spectra (600 MHz, D_2_O) of zucchini juice samples in (**a**) the aliphatic region, (**b**) the carbohydrate region, and (**c**) the aromatic region. Significant assigned signals are labelled for the detected metabolites. Abbreviations are as follows: ala, alanine; arg, arginine; asn, asparagine; asp, aspartate; eth, ethanolamine; FA, fatty acids; GABA, γ-aminobutyrate; glc, glucose; gln, glutamine; his, histidine; isoleu, isoleucine; leu, leucine; *myo*-ins, *myo*-inositol; phe, phenylalanine; thre, threonine; tyr, tyrosine; trp, tryptophan; val, valine.

A spectroscopic fingerprinting of all detectable metabolites in zucchini juice is shown in a typical ^1^H-NMR spectrum. The peaks of the main metabolites and relative expansions of significant spectral regions are labelled in Figure 2a–c. In the aliphatic region (range 0.9–3.1 ppm), it is mainly amino acids and organic acids that can be observed (Figure 2a). The most intense peaks were assigned to malate, but a great variety of other signals of lower intensity are also present. These correspond to isoleucine, leucine, valine, threonine, alanine, arginine, γ-aminobutyrate (GABA), glutamine, acetate, aspartate, asparagine and citrate. The middle region (range 3–5.5 ppm) (Figure 2b) is mostly characterized by the presence of carbohydrates with sugar resonances, such as α-β-glucose, fructose, sucrose, and *myo*-inositol, which represent the most intense peaks. In the aromatic region (5–10 ppm) (Figure 2c), together with other amino acids (histidine, phenylalanine, tyrosine, tryptophan), there are also signals attributable to nucleoside derivatives and non-aromatic compounds such as fumarate. Furthermore, the presence of phenolic compounds such as epicatechins and trigonelline can be observed. In particular, trigonelline is an alkaloid compound with important nutraceutical properties; it is produced by the methylation of nicotinic acid and present in considerable quantities in many plants [62,63]. A summary of the assigned peaks is reported on Table 1.

### 3.2. Statistical Analysis

**Figure 3:**
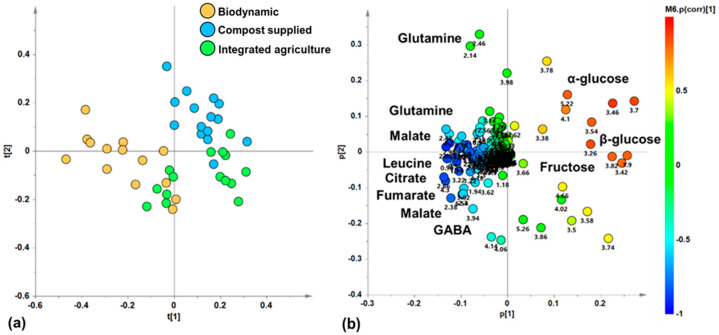
(**a**) PCA t[1]/t[2] score plot for zucchini juice samples based different cultivations (five components give R^2^X (cum) = 0.912, Q^2^ (cum) = 0.849); (**b**) loading scatter plot for the PCA model; (**a**) coloured according to the correlation scaled coefficient (p(corr) ≥ |0.5|). The colour bar associated with the plot indicates the correlation of the segregated metabolites between groups.

With the aim of discovering a possible natural grouping of zucchini juice samples, an unsupervised PCA analysis was applied to the ^1^H-NMR-spectra-derived dataset. In the resulting PCA model (R^2^X (cum) = 0.912, Q^2^(cum) = 0.849), the total variance of 91.2% was explained by five components describing the samples’ distribution in the model space (t[1], t[2], t[3], t[4] and t[5], accounting for 51%, 19.7%, 10.7%, 6.43%, and 3.36% of the total variance, respectively). In particular, the t[1]/t[2] score plot for the unsupervised model showed three clusters of zucchini juice samples (Figure 3a). Specifically, the samples of zucchini produced via biodynamic agriculture appear to be grouped in a single cluster along negative values of the principal component t[1], while the compost-supplied and integrated agriculture samples constitute two further groups along positive values of t[1]. These latter groups are further discriminated along the t[2] component, at positive (compost supplied) and negative (integrated) values. By exploring the loading scatter plot for the PCA model (Figure 3b), it was possible to identify the metabolites responsible for the observed discrimination along the t[1] and t[2] components of the PCA from their specific buckets related to the corresponding NMR signals. Zucchini samples from biodynamic agriculture, grouped at t[1] negative values, are characterized by high levels of amino acids such as leucine (0.98 ppm), glutamine (2.14, 2.42, 2.46 ppm), and GABA (2.3, 3.02 ppm), as well as organic acids such as malate (2.38, 2.7 ppm), citrate (2.54 ppm), and fumarate (6.54 ppm). On the other hand, at positive values of t[1], the samples from compost-supplied and integrated agriculture groups are characterized both by a high relative content of monosaccharides, such as α and β-glucose (loadings at 5.22 and 3.26 ppm, respectively), and fructose (4.1 ppm). In particular, samples from the compost-supplied method, grouped at positive values of t[2], are characterized by their high relative content of α and β-glucose (loadings at 5.22 and 3.26 ppm, respectively). On the other hand, the samples from integrated agriculture, grouped at negative values of t[2], are characterized by high relative content of fructose (4.1 ppm). Therefore, the unsupervised PCA analysis showed a natural separation of the zucchini samples, indicating differences in their metabolic profiles related to the different agricultural practice. These results are in agreement with those of other similar studies, in which unsupervised PCA showed a clustering of samples according to the different agricultural practices employed, confirming that the metabolomic profile is influenced by the type of crop used. In fact, in early potatoes and in table grapes, it was revelated that the metabolome depends on the crop management strategies used [11,43,45]. On the other hand, in *Brassica oleracea*, the PCA score plot did not show a clear grouping according to the cultivation [42].

**Figure 4:**
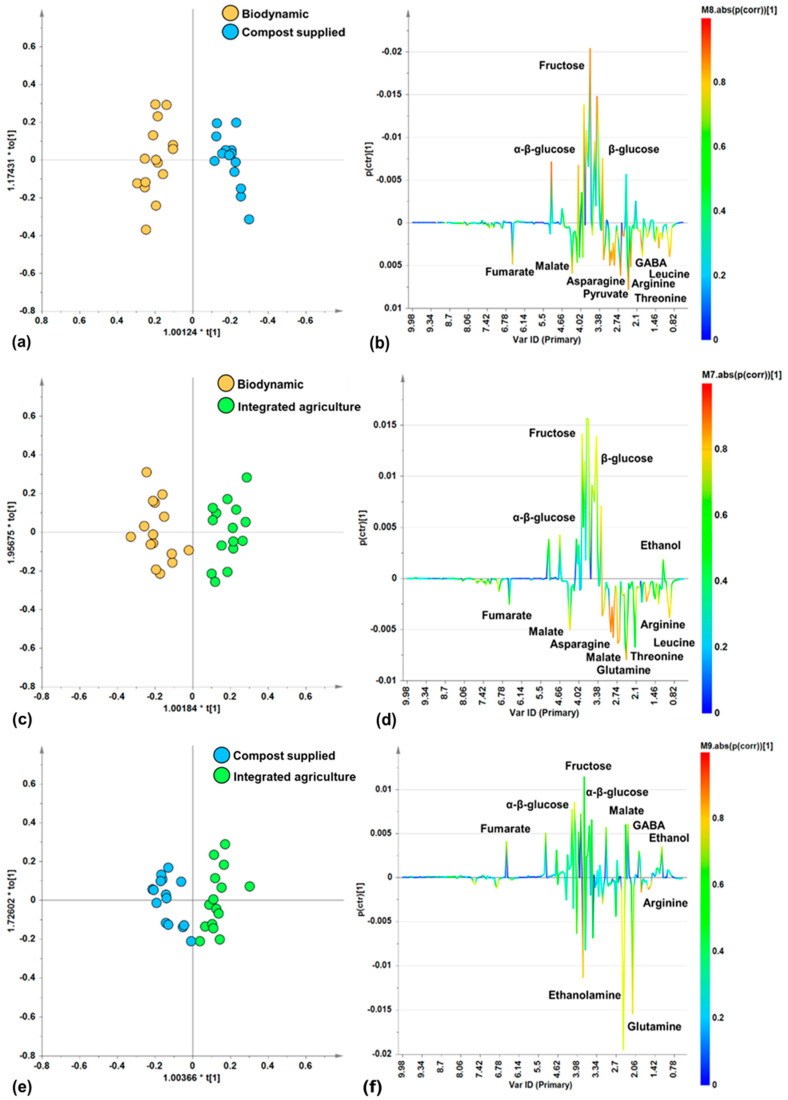
Pairwise OPLS-DA analysis comparisons between zucchini samples from different categories. (**a**) OPLS-DA score plot (30 samples) 1 + 2 + 0 components; R^2^X(cum) = 0.741, R^2^Y(cum) = 0.933, Q^2^(cum) = 0.853. (**b**) S-Line for the model (**a**). (**c**) OPLS-DA score plot (30 samples) 1 + 2 + 0 components; R^2^X(cum) = 0.725, R^2^Y(cum) = 0.888, Q^2^(cum) = 0.733. (**d**) S-Line for the model (**c**). (**e**) OPLS-DA score plot (30 samples) 1 + 2 + 0 components; R^2^X(cum) = 0.646, R^2^Y(cum) = 0.831, Q^2^(cum) = 0.684. (**f**) S-Line for the model (**e**).

In order to further investigate the specific differences in metabolites linked to the different agricultural practices (biodynamic, compost supplied, integrated agriculture), pairwise OPLS-DA supervised analyses were performed to compare the cultivation methods. The score plots of the resulting models (Figure 4a,c,e) showed, in all the pairwise comparisons (biodynamic vs. compost supplied, biodynamic vs. integrated agriculture, compost supplied vs. integrated agriculture), a good separation along the predictive component, as proved by the resulting descriptive (R^2^X, R^2^Y) and predictive (Q^2^) parameters values of the models (Table 2). Interestingly, the results of the pairwise supervised comparisons showed the best differentiation (Q^2^(cum) = 0.853) between the biodynamic and compost-supplied classes. This is followed, in order of the decreasing discrimination and predictive ability of the models, by comparisons between the biodynamic and integrated agriculture samples and the compost-supplied and integrated agriculture samples.

Moreover, by examining the corresponding S-line plots (Figure 4b,d,f) for each of the pairwise OPLS-DA models, the discriminating metabolites responsible for differentiations were identified.

In the biodynamic vs. compost-supplied comparison, the S-line plot for the OPLS-DA model (Figure 4b) showed a higher relative content of amino acids (asparagine, GABA, arginine, threonine, leucine) and organic acids (fumarate, malate, pyruvate) for biodynamic samples with respect to the compost-supplied samples which, in turn, are characterized by higher relative content of α/β-glucose and fructose.

In the biodynamic vs. integrated agriculture comparison, the S-line plot for the OPLS-DA model (Figure 4d) indicates that the biodynamic samples contain more amino acids (asparagine, glutamine, arginine, threonine, leucine) and organic acids such as fumarate and malate. On the contrary, the integrated agriculture samples have a higher relative content of α/β-glucose, fructose, and ethanol.

Lastly, in the comparison of the compost-supplied vs. integrated agriculture samples, the S-line plot for the OPLS-DA model (Figure 4f) showed that the compost-supplied samples are characterized by their higher relative content of amino acids such as ethanolamine, glutamine, and arginine. On the other hand, the integrated agriculture samples are characterized by their higher content of organic acids (fumarate, malate), the amino acid GABA, and sugars (α/β-glucose and fructose), and the constant presence of ethanol. Furthermore, the compost-supplied samples exhibited higher content of polyphenols (at 6.86 and 7.58 ppm), as observed by the broad signals in the spectra. Interestingly, as previously reported, organic zucchini fruits were characterized by significantly higher content of phenolic acids and flavonoids when compared to the integrated agriculture fruit [64].

**Figure 5:**
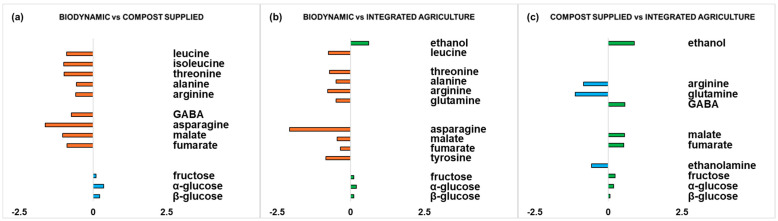
Graphic representations of the quantitative differences in discriminating metabolites for comparisons (**a**) biodynamic vs. compost supplied, (**b**) biodynamic vs. integrated agriculture, and (**c**) compost supplied vs. integrated agriculture. Strong discriminating (|p(corr)| ≥ 0.5 and VIP ≥ 1) metabolites were selected for fold change ratios. The x-axis reports significant (*p* value < 0.05) log_2_ (fold change) values.

Moreover, in order to have a complete view of the differences between the zucchini samples from the different cultivation methods, statistically significant metabolites are graphically represented (Figure 5).

For each pairwise comparison (for biodynamic vs. compost-supplied, biodynamic vs. integrated agriculture, and compost-supplied vs. integrated agriculture OPLS-DA models), quantitative estimation of the discriminating metabolite differences was performed by calculating the fold change (FC) ratios of NMR signals related to the buckets’ integrated values. Strong discriminating (|p(corr)| ≥ 0.5 and VIP ≥ 1) metabolites were selected for the calculation of FC ratios. Interestingly, statistically significant (*p* value < 0.05) differences between discriminating metabolites were obtained for each pairwise comparison.

In the biodynamic vs. compost-supplied comparison (Figure 5a), a higher significant content of amino acids (leucine, isoleucine, threonine, alanine, arginine, GABA, asparagine) and the organic acids malate and fumarate could be observed in biodynamic samples, while the compost-supplied samples exhibited a higher significant content of fructose and α/β-glucose.

Similar findings are shown in the comparison between biodynamic and integrated agriculture (Figure 5b), where the biodynamic samples resulted in a higher significant content of amino acids (leucine, threonine, alanine, arginine, glutamine, asparagine, tyrosine) and organic acids (malate and fumarate). On the other hand, the integrated agriculture samples showed, with respect to the biodynamic samples, a higher significant content of fructose and α/β-glucose and the presence of ethanol.

Finally, in the comparison between the compost-supplied and integrated agriculture samples (Figure 5c), the compost-supplied samples displayed a higher significant content of amino acids (arginine, glutamine) and ethanolamine. On the other hand, the integrated agriculture samples showed a higher significant content of the amino acid GABA, organic acids (malate and fumarate), sugars (fructose and α/β-glucose), and ethanol.

These results are consistent with other studies on the differences between samples produced using different agronomic practices. For example, Picone et al. reported that biodynamic samples of grape berries showed a lower content of sugars and a higher content of amino acids (isoleucine, GABA) and organic acids (malate), as compared to organically cultivated samples [42]. In zucchini crops, high amino acid content was described as being linked to elevated protein turnover [31]. Furthermore, for biodynamic samples, asparagine shows the highest content among the detected amino acids with respect to the other cultivation methods. Asparagine carries out an important function for nitrogen storage and transport in plants, but various mechanisms and factors that lead to the accumulation of asparagine [65]. Additionally, among other amino acids, GABA in plants has multiple functions in both non-stress and stress conditions. It influences the growth and development of plants during the crop cycle and its accumulation can help to respond to pathogens and insects [66]. Therefore, the lower GABA content in the compost-supplied samples, with respect to the biodynamic and integrated agriculture samples, could be explained by the specific probiotic supply that may generate a triple positive effect (plant growth promotion, induction of resistance to abiotic stress, induction of resistance to biotic stress). Regarding the presence of organic acids, the biosynthesis and release of organic acids are stimulated by environmental stress [67], and organic acids impact the organoleptic properties of fruits and vegetables, such as their colour, flavour and aroma [68]. Moreover, we observed a trend in the compost-supplied samples (like the biodynamic samples), in particular regarding the content of some amino acids and sugars. This key observation is interesting, since the present study focuses on the evaluation of the effect of compost supply with respect to integrated agriculture, using the biodynamic foodstuff as a benchmark. For this reason, in the present experimental design, the compost-supplied and integrated agriculture samples share the same soil. Specifically, both the biodynamic and compost-supplied samples are characterized by high arginine content. Arginine is one of the amino acids that is essential in the mechanism of resistance to fruit diseases, as it participates in various biochemical defence processes in plants [69]. Furthermore, a general increase in sugar content for the integrated agriculture products compared to the other agricultural practices can be observed; this accords with the results for table grapes described by Gallo et al. [43]. Moreover, the higher content of fructose in the integrate zucchini samples is in agreement with the findings of Lucarini et al. in the comparison of organic and conventional *Brassica oleracea* [44]. In our case, the higher content of sugars observed in the integrated agriculture zucchini samples could explain the increased ethanol production, compared to the biodynamic and compost-supplied samples. In fact, more ethanol was observed in the integrated agriculture samples in all the comparisons. Ethanol is an important signal of changes in metabolic profiles [70], being the consequence of sugar fermentation during fruit ripening [71]. In another study, where the biodynamic samples were grown in the same soil used for the biodynamic samples in this research, higher ethanol levels, ascribable to a possible increased yeast presence, were found in the biodynamic samples with respect to organic and integrated table grapes, where the sugar content did not constitute a limiting factor [11].

### 3.3. Element Content in Different Zucchini Samples

**Figure 6:**
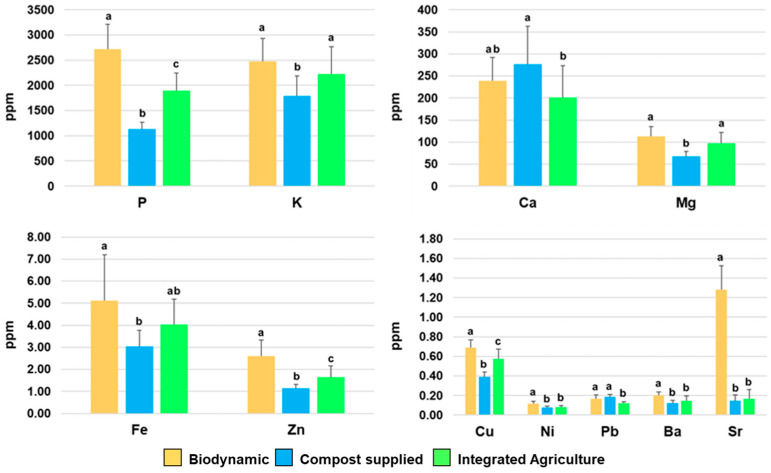
Concentration of significant elements in zucchini samples expressed in ppm. Data were obtained using the ICP-AES technique and are expressed as means ± standard deviations. Ca: calcium; P, phosphorus; Cu, copper; Fe, iron; Ni, nickel; Pb, lead; K, potassium; Mg, magnesium; Ba, barium; Sr, strontium; Zn, zinc. Bars represent standard errors. Different letters indicate statistical significance at *p* < 0.05.

Furthermore, the determination of the concentration of significant elements (Ca, P, Cu, Fe, Ni, Pb, K, Mg, Ba, Sr, and Zn), (*p* < 0.05), was carried out via ICP-AES analysis (Figure 6) (Table S3). The averaged results obtained using the elemental data for each class of the investigated samples appear almost parallel to the trend observed when applying MVA to the NMR data set (Figure S7). Moreover, it should be noted that, in this study, only the integrated and compost-supplied products share the same soil, with the Demeter-certified product used as a benchmark originating in a different area. In particular, the biodynamic samples show specific differences with respect to the compost-supplied and integrated products; these are possibly related to both the cultivation method and the soil features. Interestingly, although biodynamic agriculture focuses on a limited or absent external supply to the soil, the general trend for P, K, and Mg (which are among the most abundant detected elements) shows a consistent increase for biodynamic cultivation products with respect to the others. Moreover, in the biodynamic products, Ca also increases as compared to integrated agriculture cultivations, but it shows slightly lower values if compared to the compost-supplied variety. A similar behaviour is also observed for the less abundant elements, in particular, for Fe, Zn, Cu and Sr, which exhibit higher concentrations in the biodynamic samples with respect to compost-supplied and integrated zucchini samples. Further results that could be highlighted are the relatively close, although smaller (except for Ca), concentration values of the most abundant detected elements (P, K, Ca, Mg), together with Fe, Zn, and Cu, obtained for the compost-supplied products with respect to the integrated agriculture products. This finding may indicate that, at least in the present case, the compost supply does not significantly decrease the key element accumulation in the products with respect to integrated agriculture farming. Finally, the roughly similar concentrations observed for the remaining elements (Ni, Pb, Ba, and Sr) only show a limited significant increased accumulation of Pb for the compost-supplied group with respect to integrated products, which remain comparable with the results of the biodynamic samples. These results are generally aligned with those of other studies focused on the differences in key nutrient content between organic and integrated agriculture methods. In particular, the higher content of specific key elements, such as Ca, K, Mg, Na, P, and S, in organic fresh vegetables (cabbage, kohlrabi, Brussels sprouts, beetroot, carrot, potato, and onion) with respect to those produced conventionally, was observed [72]. On the other hand, in leeks, only K was significantly influenced by the management system, showing a higher concentration in organic agriculture with respect to conventional system [73]. In tomatoes, the analysis of the element content showed significant differences in Ca, Na, Fe, and Zn concentrations between biodynamic and conventional tomatoes, while no important differences were observed between biodynamic and organic ones [74]. Furthermore, in potatoes, organic cultivation has produced tubers with mineral content different to that of conventional crops [75].

For all other elements analysed (Cd, Mn, Cr, Co, Be, Li, Te, Tl, and As), the results indicate concentrations below the sensitivity limit of the used detection method. Changes in the content of elements (macronutrients and micronutrients) related to the use of fertilizers have already been reported for agricultural products [76]. Moreover, it was observed that organic farming led to improved soil health conditions [77]. On the other hand, integrated practices and, specifically, land tillage on both organic and integrated agriculture farms degraded the soil organic matter by reducing mineral absorption [77]. Meanwhile, microbial inoculants and compost could increase the content of crop micronutrients, reconstituting soil organic matter, on both organic and integrated agriculture farms [77]. In this respect, our findings indicate that compost supply is a viable alternative to integrated management for improving key nutrient element uptake in food production. Furthermore, our study showed clear results in terms of the superior performances obtained in term of key elements uptake with biodynamic cultivation, and possibly ascribable to its specific microbiota profile [78]. Therefore, in the present case, the use of a specific compost as a biofertilizer was shown to favour the transition to the quality standards of Demeter production, significantly improving crop sustainability [79].

## 4. Conclusions

Our findings demonstrated that the metabolic profiles of zucchini samples, although they were from the same cultivar *Vitulia*, are significantly influenced by different agricultural practices. Our results showed that the samples from the plots managed using biofertilizer from compost showed similar behaviour to the samples managed according to the principles of Demeter biodynamic certification, with higher content of specific amino acids, such as arginine, and lower content of sugars than the samples from integrated farming. Our research suggests that the use of a specific compost as biofertilizer could affect the metabolic profiles of the analysed samples, exhibiting significant discriminating metabolites (sugars, amino acids, and organic acids) that could represent a possible marker combination, which is also related to the quality of zucchini. Moreover, the results of the elemental analysis indicate compost supply to be a viable alternative to integrated management for improving key nutrient element uptake in agriculture crops. Therefore, in the present case, the use of a specific compost as a biofertilizer was shown to favour the transition to the quality standards of Demeter certification, significantly improving crop sustainability.

## Figures and Tables

**Table 1:** ^1^H and ^13^C chemical shifts of the assigned metabolites of the zucchini juice sample.

Metabolites	Assignment	^1^H ppm	Multiplicity *; J (Hz)	^13^C ppm
Valine	γ′-C*H*_3_ γ-C*H*_3_ β-C*H*	1.05 0.99 2.27	d [7.0] d [7.0] m	20 19.3
Isoleucine	γ-C*H*_3_ δ-C*H*_3_	1.01 0.94	d [7.2] t [7.36]	19 13.08
Leucine	γ-C*H*_3_ γ′-C*H*_3_	0.96 0.97	d d	23 23
Ethanol	β-C*H*_3_ α-C*H*_2_	1.17 3.66	t [7.26] q	
Threonine	γ-C*H*_3_ β-C*H*	1.33 4.25	d [6.28] m	22
Alanine	β-C*H*_3_ α-C*H* -COO*H*	1.47 3.77	D [7.3] q	19.3 179.3
Arginine	γ-C*H*_2_ β-C*H*_2_	1.72 1.90	m m	30 26.9
GABA	β-C*H*_2_ α-C*H*_2_ γ-C*H*_2_ -COO*H*	1.90 2.30 3.0	m t [7.58] t [7.4]	26.63 37.05 42.29 184.6
Acetate	β-C*H*_3_	1.92	s	
Glutamine	γ-C*H*_2_ β-C*H*_2_	2.44 (m) 2.14 (m)	m m	33.84 29
Malate	α-C*H* β′-C*H* β-C*H* -COO*H*	4.31 2.70 2.40	dd [10; 3] dd [15.1; 3.15] dd [15.2; 10]	72.6 44 44 183
Citrate	CH_2_	2.54	d	
Aspartate	β-C*H* β′-C*H*	2.80 2.64	dd [17.9; 3.7] dd	39.4 39.4
Asparagine	β-C*H* β′-C*H* COO*H*	2.94 [16.7; 4.2] 2.83 [16.6; 7.4]	dd dd	37.4 37.4 176
Ethanolamine	C*H*_2_-NH_2_	3.12	t	44
Choline	N(C*H*_3_)_3_	3.20	s	56
*Myo*-inositol	C2*H* C4*H* C5*H*	4.07 3.62 3.28	t t t	77
β-glucose	C1*H* C3*H* C2*H*	4.64 3.48 3.26	d[8.3] dd t [8.0]	98 77
α-glucose	C1*H* C2*H*	5.23 3.51	d [3.74] t	95
Sucrose	G1*H* G2*H*	5.38 3.55	d [3.8] dd	
α/β-fructofuranose	C3H C5H	4.11 3.84		78 83
β-fructopyranose	C5H CH_2_-6,6′ CH_2_-1,1′	4.02 3.70 3.72; 3.58		71 63.4
Uridine	C5*H*, ring C6*H*, ring	7.90 5.91	d [8.84] d [7.97]	
Adenosine	C2*H*, ring C*H*8, ring C1′H, ribose	8.35 8.27 6.07	s s d	
Fumarate	α,β-C=C -COO*H*	6.52	s	138 178.4
Uracil	C*H*5, ring C*H*6, ring	7.53 5.8	d d	
Trigonelline	N-C*H* C*H*-3,5 C*H*-5	9.12 8.85 8.10	s t dd	
Histidine	C*H*2-ring C*H*4-ring	8.0 7.14	s s	
Tryptophan	C*H*4, ring C*H*7, ring C*H*5, ring	7.72 7.55 7.19	d d d	131
Tyrosine	C*H*2, C*H*6, ring C*H*3, C*H*5 ring	7.19 6.90	d [8.3] d [8.0]	133 118
Phenylalanine	C*H*4, ring C*H*3,5-ring C*H*2,6-ring	7.38 7.43 7.33	m m m	132 132.3

* Letters in parentheses indicate the peak multiplicities: s, singlet; d, doublet; t, triplet; dd, doublet of doublet; m, multiplet.

**Table 2:** Descriptive and predictive model parameters used to assess the robustness of the pairwise OPLS-DA analysis models. One predictive and two orthogonal components were considered for each model.

Pairwise Comparison	R^2^X (cum)	R^2^Y (cum)	Q^2^ (cum)	Permutation Test R^2^; Q^2^ Intercepts	CV-ANOVA	Fisher’s Probability *
Biodynamic vs. Compost Supplied	0.741	0.933	0.853	0.24; −0.522	1.66 × 10^−8^	2.7 × 10^−8^
Biodynamic vs. Integrated Agriculture	0.725	0.888	0.733	0.265; −0.685	1.21 × 10^−5^	2.7 × 10^−8^
Compost Supplied vs. Integrated Agriculture	0.646	0.831	0.684	0.269; −0.536	7.40 × 10^−5^	6.4 × 10^−9^

* From Fisher’s exact test displayed in the misclassification table (SIMCA-P software).

## Data Availability

Data are contained within the article and in the Supplementary Materials. Further inquiries can be directed to the corresponding author.

## Supplementary Materials

The following supporting information can be downloaded at: https://www.mdpi.com/article/10.3390/foods14060919/s1, Figure S1: Photo of cultivar *Vitulia* (Syngenta CV 2832) zucchini described in this study. From https://agronotizie.imagelinenetwork.com/agronomia/2010/05/11/zucchino-otto-nuove-varieta-targate-syngenta/9325 (accessed on 5 March 2025). Figure S2: Cultivation soil for zucchini samples. They refer to two collaborating specialised Italian farms, located in the south of Italy (Apulian Region). Lacalamita Rosa (Castellaneta, Taranto, Italy) provided the biodynamic farming while Tenuta Pinto (Mola di Bari, Bari, Italy) was involved in compost-supplied and integrated agriculture trials. (a) Biodynamic soil; (b) compost-supplied soil; (c) integrated agriculture soil. Figure S3: ^1^H-^1^H cosy spectrum of a representative zucchini juice sample. Figure S4: ^1^H-J-resolved spectrum of a representative zucchini juice sample. Figure S5: ^1^H-^13^C hsqc 2D spectrum of a representative zucchini juice sample. Expansions in the 0.5–3; 3–4.5; and 6–9 spectral regions are represented. Figure S6: ^1^H-^13^C hmbc 2D spectrum of a representative zucchini juice sample. Expansions in the −0.5–4.5 and 5–7.5 spectral regions are represented. Figure S7: (a) PCA score plot for all measured elements. (b) Loading scatter plot for the model. Table S1: List of biodynamic preparations and their effects. Table S2: Technical data sheet of compost-supplied “Bio Vegetal”. Bio Vegetal is a biofertilizer with organic substances and 200 types of live microorganisms. Table S3: Concentrations of significant elements in zucchini samples expressed in ppm. Data were obtained using the ICP-AES technique and are expressed as means ± standard deviations. Results are presented as the mean (±standard deviation) of different measurements for each of the three cultivation methods. Ca: calcium; P, phosphorus; Cu, copper; Fe, iron; Ni, nickel; Pb, lead; K, potassium; Mg, magnesium; Ba, barium; Sr, strontium; Zn, zinc.

## Abbreviations

The following abbreviations are used in this manuscript:

NMR	Nuclear magnetic resonance
ICP-AES	Inductively coupled plasma atomic emission spectroscopy
MVA	Multivariate statistical analysis
PCA	Principal component analysis
OPLS-DA	Orthogonal partial least squares discriminant analysis

## References

[1] Martínez-Valdivieso D., Font R., Fernández-Bedmar Z., Merinas-Amo T., Gómez P., Alonso-Moraga Á., Del Río-Celestino M. (2017). Role of Zucchini and Its Distinctive Components in the Modulation of Degenerative Processes: Genotoxicity, Anti-Genotoxicity, Cytotoxicity and Apoptotic Effects. Nutrients.

[2] Iswaldi I., Gómez-Caravaca A.M., Lozano-Sánchez J., Arráez-Román D., Segura-Carretero A., Fernández-Gutiérrez A. (2013). Profiling of Phenolic and Other Polar Compounds in Zucchini (*Cucurbita pepo* L.) by Reverse-Phase High-Performance Liquid Chromatography Coupled to Quadrupole Time-of-Flight Mass Spectrometry. Food Res. Int..

[3] Cagliani L.R., Consonni R. (2024). Monitoring the Metabolite Content of Seasoned Zucchinis during Storage by NMR-Based Metabolomics. Heliyon.

[4] Sofi F., Macchi C., Abbate R., Gensini G.F., Casini A. (2013). Mediterranean Diet and Health. BioFactors.

[5] Tejada L., Buendía-Moreno L., Villegas A., Cayuela J.M., Bueno-Gavilá E., Gómez P., Abellán A. (2020). Nutritional and Sensorial Characteristics of Zucchini (*Cucurbita pepo* L.) as Affected by Freezing and the Culinary Treatment. Int. J. Food Prop..

[6] Hounsome N., Hounsome B., Tomos D., Edwards-Jones G. (2008). Plant Metabolites and Nutritional Quality of Vegetables. J. Food Sci..

[7] Dernini S., Berry E., Serra-Majem L., La Vecchia C., Capone R., Medina F., Aranceta-Bartrina J., Belahsen R., Burlingame B., Calabrese G. (2017). Med Diet 4.0: The Mediterranean Diet with Four Sustainable Benefits. Public. Health Nutr..

[8] Döring J., Frisch M., Tittmann S., Stoll M., Kauer R. (2015). Growth, Yield and Fruit Quality of Grapevines under Organic and Biodynamic Management. PLoS ONE.

[9] Romero H., Pott D.M., Vallarino J.G., Osorio S. (2021). Metabolomics-Based Evaluation of Crop Quality Changes as a Consequence of Climate Change. Metabolites.

[10] Rouphael Y., Cardarelli M., Bassal A., Leonardi C., Giuffrida F., Colla G. (2012). Vegetable Quality as Affected by Genetic, Agronomic and Environmental Factors. J. Food Agric. Environ..

[11] Colì C.S., Girelli C.R., Cesari G., Hussain M., Fanizzi F.P. (2024). Biodynamic, Organic and Integrated Agriculture Effects on Cv. Italia Table Grapes Juice, over a 3-Year Period Experiment: An 1H NMR Spectroscopy-Based Metabolomics Study. Chem. Biol. Technol. Agric..

[12] Turinek M., Grobelnik-Mlakar S., Bavec M., Bavec F. (2009). Biodynamic Agriculture Research Progress and Priorities. Renew. Agric. Food Syst..

[13] Pergola M., Persiani A., Palese A.M., Di Meo V., Pastore V., D’Adamo C., Celano G. (2018). Composting: The Way for a Sustainable Agriculture. Appl. Soil. Ecol..

[14] Steiner R. (2014). Impulsi Scientifico-Spirituali per IL Progresso Dell’Agricoltura: Corso Sull’Agricoltura.

[15] Santoni M., Ferretti L., Migliorini P., Vazzana C., Pacini G.C. (2022). A Review of Scientific Research on Biodynamic Agriculture. Org. Agric..

[16] Demeter—Biodynamic Federation Demeter International. https://demeter.net/.

[17] FiBL IFOMA-Organics International (2020). The World of Organic Agriculture: Statistics & Emerging Trends 2020.

[18] Biodynamic Preparations (2021). Demeter International. https://www.demeter.net/wp-content/uploads/2021/11/BFDI_Biodynamic-Preparation-Manual-Plant-cultivation_2021_EN.pdf.

[19] Regolamento (UE) 2018/Del Parlamento Europeo e Del Consiglio, Del 30 Maggio 2018, Relativo Alla Produzione Biologica e All’etichettatura Dei Prodotti Biologici e Che Abroga Il Regolamento (CE) n.834/2007 Del Consiglio. https://eur-lex.europa.eu/legal-content/IT/TXT/PDF/?uri=CELEX:32018R0848.

[20] Agnew J.M., Leonard J.J. (2003). The Physical Properties of Compost. Compost. Sci. Util..

[21] Martínez-Blanco J., Lazcano C., Christensen T.H., Muñoz P., Rieradevall J., Møller J., Antón A., Boldrin A. (2013). Compost Benefits for Agriculture Evaluated by Life Cycle Assessment. A Review. Agron. Sustain. Dev..

[22] Azim K., Soudi B., Boukhari S., Perissol C., Roussos S., Thami Alami I. (2018). Composting Parameters and Compost Quality: A Literature Review. Org. Agric..

[23] Cao X., Williams P.N., Zhan Y., Coughlin S.A., McGrath J.W., Chin J.P., Xu Y. (2023). Municipal Solid Waste Compost: Global Trends and Biogeochemical Cycling. Soil Environ. Health.

[24] Bio Vegetal (2017). Tersan Puglia. https://www.tersan.it/bio-vegetal/.

[25] Il Compostaggio (2016). Tersan Puglia. https://www.tersan.it/il-processo-di-compostaggio-tersan/.

[26] Çakmakçı R., Salık M.A., Çakmakçı S. (2023). Assessment and Principles of Environmentally Sustainable Food and Agriculture Systems. Agriculture.

[27] Boschiero M., De Laurentiis V., Caldeira C., Sala S. (2023). Comparison of Organic and Conventional Cropping Systems: A Systematic Review of Life Cycle Assessment Studies. Environ. Impact Assess. Rev..

[28] Wright J. (2022). A Revitalisation of European Farming and the Promise of the Biodynamic Worldview. Chem. Biol. Technol. Agric..

[29] Azarbad H. (2022). Conventional vs. Organic Agriculture–Which One Promotes Better Yields and Microbial Resilience in Rapidly Changing Climates?. Front. Microbiol..

[30] Sumberg J., Giller K.E. (2022). What Is ‘Conventional’ Agriculture?. Glob. Food Secur..

[31] Abreu A.C., Aguilera-Sáez L.M., Peña A., García-Valverde M., Marín P., Valera D.L., Fernández I. (2018). NMR-Based Metabolomics Approach To Study the Influence of Different Conditions of Water Irrigation and Greenhouse Ventilation on Zucchini Crops. J. Agric. Food Chem..

[32] Girelli C.R., Accogli R., Del Coco L., Angilè F., De Bellis L., Fanizzi F.P. (2018). 1H-NMR-Based Metabolomic Profiles of Different Sweet Melon (*Cucumis melo* L.) Salento Varieties: Analysis and Comparison. Food Res. Int..

[33] Vignoli A., Ghini V., Meoni G., Licari C., Takis P.G., Tenori L., Turano P., Luchinat C. (2019). High-Throughput Metabolomics by 1D NMR. Angew. Chem. Int. Ed..

[34] Al-Akl N.S., Khalifa O., Ponirakis G., Parray A., Ramadan M., Khan S., Chandran M., Ayadathil R., Elsotouhy A., Own A. (2024). Untargeted Metabolomic Profiling Reveals Differentially Expressed Serum Metabolites and Pathways in Type 2 Diabetes Patients with and without Cognitive Decline: A Cross-Sectional Study. Int. J. Mol. Sci..

[35] Serio F., Girelli C.R., Acito M., Imbriani G., Sabella E., Moretti M., Fanizzi F.P., Valacchi G. (2024). Preliminary Characterization of “Salice Salentino” PDO Wines from Salento (South Italy) Negroamaro Grapes: NMR-Based Metabolomic and Biotoxicological Analyses. Foods.

[36] Papadia P., Del Coco L., Muzzalupo I., Rizzi M., Perri E., Cesari G., Simeone V., Mondelli D., Schena F.P., Fanizzi F.P. (2011). Multivariate Analysis of ^1^H-NMR Spectra of Genetically Characterized Extra Virgin Olive Oils and Growth Soil Correlations. J. Am. Oil Chem. Soc..

[37] Girelli C.R., Serio F., Accogli R., Angilè F., De Donno A., Fanizzi F.P. (2021). First Insight into Nutraceutical Properties of Local Salento Cichorium Intybus Varieties: NMR-Based Metabolomic Approach. Int. J. Environ. Res. Public. Health.

[38] Sobolev A., Mannina L., Proietti N., Carradori S., Daglia M., Giusti A., Antiochia R., Capitani D. (2015). Untargeted NMR-Based Methodology in the Study of Fruit Metabolites. Molecules.

[39] Girelli C.R., De Pascali S.A., Del Coco L., Fanizzi F.P. (2016). Metabolic Profile Comparison of Fruit Juice from Certified Sweet Cherry Trees (*Prunus avium* L.) of Ferrovia and Giorgia Cultivars: A Preliminary Study. Food Res. Int..

[40] Girelli C.R., Papadia P., Pagano F., Miglietta P.P., Fanizzi F.P., Cardinale M., Rustioni L. (2023). Metabolomic NMR Analysis and Organoleptic Perceptions of Pomegranate Wines: Influence of Cultivar and Yeast on the Product Characteristics. Heliyon.

[41] Savorani F., Rasmussen M.A., Mikkelsen M.S., Engelsen S.B. (2013). A Primer to Nutritional Metabolomics by NMR Spectroscopy and Chemometrics. Food Res. Int..

[42] Picone G., Trimigno A., Tessarin P., Donnini S., Rombolà A.D., Capozzi F. (2016). ^1^H NMR Foodomics Reveals That the Biodynamic and the Organic Cultivation Managements Produce Different Grape Berries (*Vitis vinifera* L. Cv. Sangiovese). Food Chem..

[43] Gallo V., Mastrorilli P., Cafagna I., Nitti G.I., Latronico M., Longobardi F., Minoja A.P., Napoli C., Romito V.A., Schäfer H. (2014). Effects of Agronomical Practices on Chemical Composition of Table Grapes Evaluated by NMR Spectroscopy. J. Food Compos. Anal..

[44] Lucarini M., Di Cocco M.E., Raguso V., Milanetti F., Durazzo A., Lombardi-Boccia G., Santini A., Delfini M., Sciubba F. (2020). NMR-Based Metabolomic Comparison of *Brassica Oleracea* (Var. Italica): Organic and Conventional Farming. Foods.

[45] Pacifico D., Casciani L., Ritota M., Mandolino G., Onofri C., Moschella A., Parisi B., Cafiero C., Valentini M. (2013). NMR-Based Metabolomics for Organic Farming Traceability of Early Potatoes. J. Agric. Food Chem..

[46] Hussain M., Girelli C.R., Verweire D., Oehl M.C., Avendaño M.S., Scortichini M., Fanizzi F.P. (2023). ^1^H-NMR Metabolomics Study after Foliar and Endo-Therapy Treatments of *Xylella fastidiosa* Subsp. Pauca Infected Olive Trees: Medium Time Monitoring of Field Experiments. Plants.

[47] Van Den Berg R.A., Hoefsloot H.C., Westerhuis J.A., Smilde A.K., Van Der Werf M.J. (2006). Centering, Scaling, and Transformations: Improving the Biological Information Content of Metabolomics Data. BMC Genom..

[48] Eriksson L., Byrne T., Johansson E., Trygg J., Vikström C. (2013). Multi—And Megavariate Data Analysis Basic Principles and Applications.

[49] Jackson J.E. (2005). A User’s Guide to Principal Components.

[50] Kettaneh N., Berglund A., Wold S. (2005). PCA and PLS with Very Large Data Sets. Comput. Stat. Data Anal..

[51] Chen Y., Li E.-M., Xu L.-Y. (2022). Guide to Metabolomics Analysis: A Bioinformatics Workflow. Metabolites.

[52] Eriksson L., Rosén J., Johansson E., Trygg J. (2012). Orthogonal PLS (OPLS) Modeling for Improved Analysis and Interpretation in Drug Design. Mol. Inform..

[53] Wold S., Eriksson L., Trygg J., Kettaneh N. (2004). The PLS Method—Partial Least Squares Projections to Latent Structures—And Its Applications in Industrial RDP (Research, Development, and Production). COMPSTAT 2004, Proceedings of the Computational Statistics, 16th Symposium of IASC, Prague, Czech Republic, January 2004.

[54] Trygg J., Wold S. (2002). Orthogonal Projections to Latent Structures (O-PLS). J. Chemom..

[55] Wheelock Å.M., Wheelock C.E. (2013). Trials and Tribulations of ‘omics Data Analysis: Assessing Quality of SIMCA-Based Multivariate Models Using Examples from Pulmonary Medicine. Mol. Biosyst..

[56] Leleu G., Garcia L., Homobono Brito De Moura P., Da Costa G., Saucier C., Richard T. (2025). Cork Impact on Red Wine Aging Monitoring through ^1^H NMR Metabolomics: A Comprehensive Approach. Food Res. Int..

[57] Angilè F., Vivaldi G.A., Girelli C.R., Del Coco L., Caponio G., Lopriore G., Fanizzi F.P., Camposeo S. (2022). Treated Unconventional Waters Combined with Different Irrigation Strategies Affect 1H NMR Metabolic Profile of a Monovarietal Extra Virgin Olive Oil. Sustainability.

[58] Cocchi M., Biancolillo A., Marini F. (2018). Chemometric Methods for Classification and Feature Selection. Compr. Anal. Chem..

[59] Scortichini M., Chen J., de Caroli M., Dalessandro G., Pucci N., Modesti V., L’aurora A., Petriccione M., Zampella L., Mastrobuoni F. (2018). A Zinc, Copper and Citric Acid Biocomplex Shows Promise for Control of *Xylella fastidiosa* Subsp. *pauca* in Olive Trees in Apulia Region (Southern Italy). Phytopathol. Mediterr..

[60] Scortichini M., Migoni D., Angilè F., Del Coco L., Girelli C.R., Zampella L., Mastrobuoni F., Fanizzi F.P. (2019). *Xylella fastidiosa* Subsp. Pauca on Olive in Salento (Southern Italy). Phytopathol. Mediterr..

[61] MetaboAnalyst. https://www.metaboanalyst.ca/MetaboAnalyst/ModuleView.xhtml.

[62] Liang Y., Dai X., Cao Y., Wang X., Lu J., Xie L., Liu K., Li X. (2023). The Neuroprotective and Antidiabetic Effects of Trigonelline: A Review of Signaling Pathways and Molecular Mechanisms. Biochimie.

[63] Ashihara H. (2008). Trigonelline (*N*-Methylnicotinic Acid) Biosynthesis and Its Biological Role in Plants. Nat. Prod. Commun..

[64] Kopczyńska K., Kazimierczak R., Średnicka-Tober D., Barański M., Wyszyński Z., Kucińska K., Perzanowska A., Szacki P., Rembiałkowska E., Hallmann E. (2020). The Profile of Selected Antioxidants in Two Courgette Varieties from Organic and Conventional Production. Antioxidants.

[65] Lea P.J., Sodek L., Parry M.A.J., Shewry P.R., Halford N.G. (2007). Asparagine in Plants. Ann. Appl. Biol..

[66] Ramos-Ruiz R., Martinez F., Knauf-Beiter G. (2019). The Effects of GABA in Plants. Cogent Food Agric..

[67] Panchal P., Miller A.J., Giri J. (2021). Organic Acids: Versatile Stress-Response Roles in Plants. J. Exp. Bot..

[68] Chahardoli A., Jalilian F., Memariani Z., Farzaei M.H., Shokoohinia Y. (2020). Analysis of Organic Acids. Recent Advances in Natural Products Analysis.

[69] Modolo L.V., Augusto O., Almeida I.M.G., Pinto-Maglio C.A.F., Oliveira H.C., Seligman K., Salgado I. (2006). Decreased Arginine and Nitrite Levels in Nitrate Reductase-Deficient Arabidopsis Thaliana Plants Impair Nitric Oxide Synthesis and the Hypersensitive Response to Pseudomonas Syringae. Plant Sci..

[70] Biais B., Allwood J.W., Deborde C., Xu Y., Maucourt M., Beauvoit B., Dunn W.B., Jacob D., Goodacre R., Rolin D. (2009). ^1^H NMR, GC−EI-TOFMS, and Data Set Correlation for Fruit Metabolomics: Application to Spatial Metabolite Analysis in Melon. Anal. Chem..

[71] Dudley R. (2004). Ethanol, Fruit Ripening, and the Historical Origins of Human Alcoholism in Primate Frugivory. Integr. Comp. Biol..

[72] Popović-Djordjević J.B., Kostić A.Ž., Rajković M.B., Miljković I., Krstić Đ., Caruso G., Siavash Moghaddam S., Brčeski I. (2022). Organically vs. Conventionally Grown Vegetables: Multi-Elemental Analysis and Nutritional Evaluation. Biol. Trace Elem. Res..

[73] Golubkina N.A., Seredin T.M., Antoshkina M.S., Kosheleva O.V., Teliban G.C., Caruso G. (2018). Yield, Quality, Antioxidants and Elemental Composition of New Leek Cultivars under Organic or Conventional Systems in a Greenhouse. Horticulturae.

[74] D’Evoli L., Lucarini M., Del Pulgar J.S., Aguzzi A., Gabrielli P., Gambelli L., Lombardi-Boccia G. (2016). Phenolic Acids Content and Nutritional Quality of Conventional, Organic and Biodynamic Cultivations of the Tomato CXD271BIO Breeding Line (*Solanum lycopersicum* L.). Food Nutr. Sci..

[75] Lombardo S., Pandino G., Mauromicale G. (2014). The Mineral Profile in Organically and Conventionally Grown “Early” Crop Potato Tubers. Sci. Hortic..

[76] Wang Z., Li S., Malhi S. (2008). Effects of Fertilization and Other Agronomic Measures on Nutritional Quality of Crops. J. Sci. Food Agric..

[77] Montgomery D.R., Biklé A. (2021). Soil Health and Nutrient Density: Beyond Organic vs. Conventional Farming. Front. Sustain. Food Syst..

[78] Calabrese A., Mandrelli L., Loi E., Blonda M. (2020). Chemical and Microbiological Characterization of Soil under Different Agronomical Use and Practical: First Focus on Nitrogen Cycles. IOSR J. Biotechnol. Biochem..

[79] Platis D.P., Anagnostopoulos C.D., Tsaboula A.D., Menexes G.C., Kalburtji K.L., Mamolos A.P. (2019). Energy Analysis, and Carbon and Water Footprint for Environmentally Friendly Farming Practices in Agroecosystems and Agroforestry. Sustainability.

