# Supplementary material for: 1H-NMR-Based Metabolomic Profiles of Zucchini (Cucurbita pepo L.) Grown with Different Agricultural Practices for Sustainable Crop Production
Source: Foods. 2025 Mar 7;14(6):919. doi: 10.3390/foods14060919 (PMC11941092; doi:10.3390/foods14060919)
Supplement: Supplementary file 1 [file foods-14-00919-s001.zip › foods-3488314-supplementary.pdf]

Supplementary material

^{1}H-NMR-based metabolomic profiles of zucchini (*Cucurbita pepo* L.) grown with different agricultural practices for sustainable crop production

Miriana Carla Fazzi [1], Chiara Roberta Girelli [1], Danilo Migoni [1], Beatrice Fracasso[1], Gianluigi Cesari [2] and Francesco Paolo Fanizzi [1, *]

[1] Department of Biological and Environmental Sciences and Technology, University of Salento, 73100 Lecce, Italy; mirianacarla.fazzi@unisalento.it; chiara.girelli@unisalento.it; danilo.migoni@unisalento.it; beatrice.fracasso@unisalento.it

[2] CIHEAM-Bari, Via Ceglie 9, 70010 Valenzano (Bari), Italy; cesari@iamb.it

* Correspondence: fp.fanizzi@unisalento.it

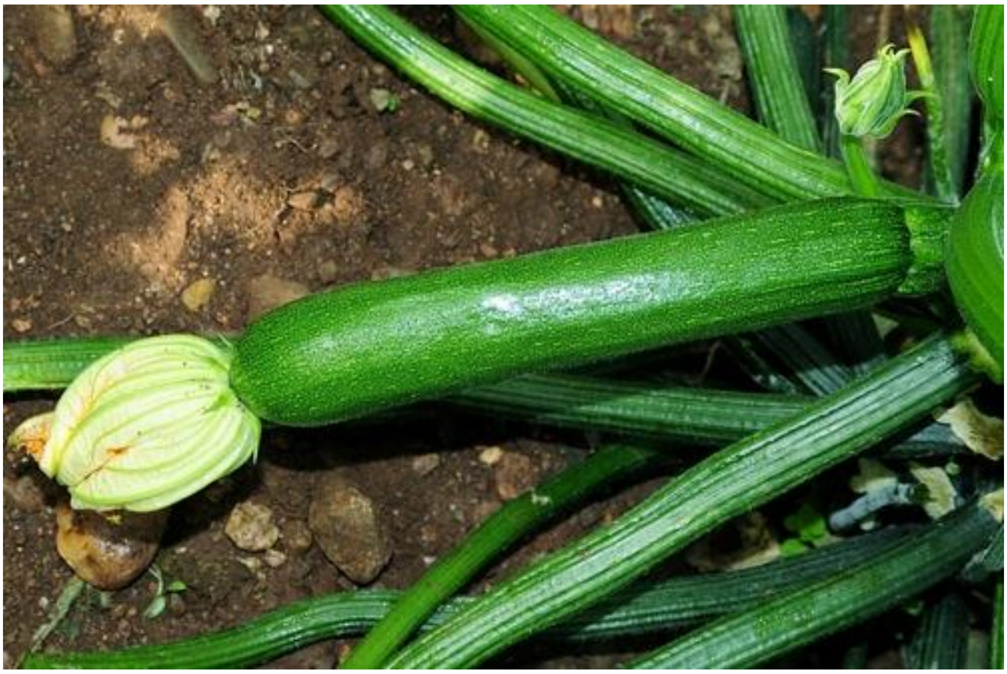

Figure S1. Photo of cultivar *Vitulia* (Syngenta CV 2832) zucchini described in this study. (From https://agronotizie.imagelinenetwork.com/agronomia/2010/05/11/zucchino-otto-nuove-varieta-targate-syngenta/9325)

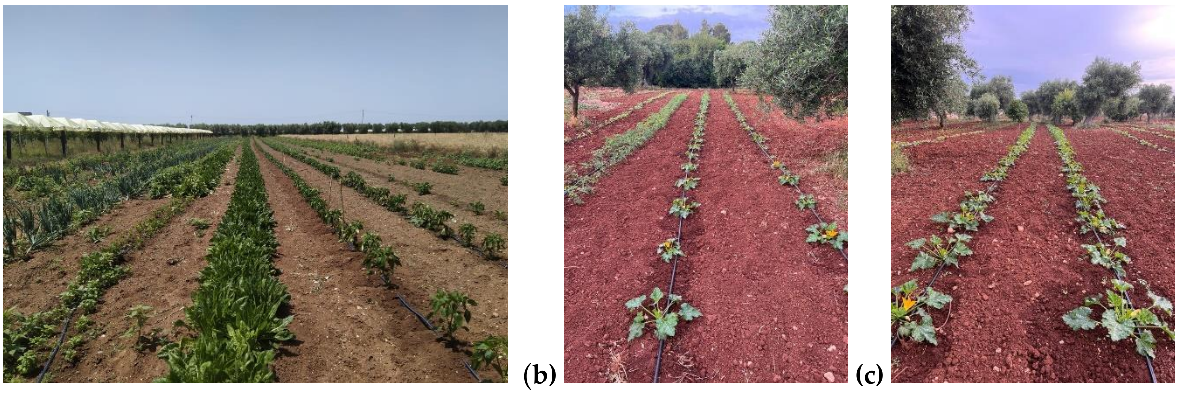

(a) **(b)** **(c)**

Figure S2. Cultivations soil for zucchini samples. They refer to two collaborating specialised Italian Farms, placed in the south of Italy (Apulian Region). Lacalamita Rosa (Castellaneta, Taranto, Italy) provided the biodynamic farming while Tenuta Pinto, (Mola di Bari, Bari, Italy) was involved in compost supplied and integrated agriculture trials. **a)** Biodynamic soil; **b)** Compost supplied soil; **c)** Integrated agriculture soil.

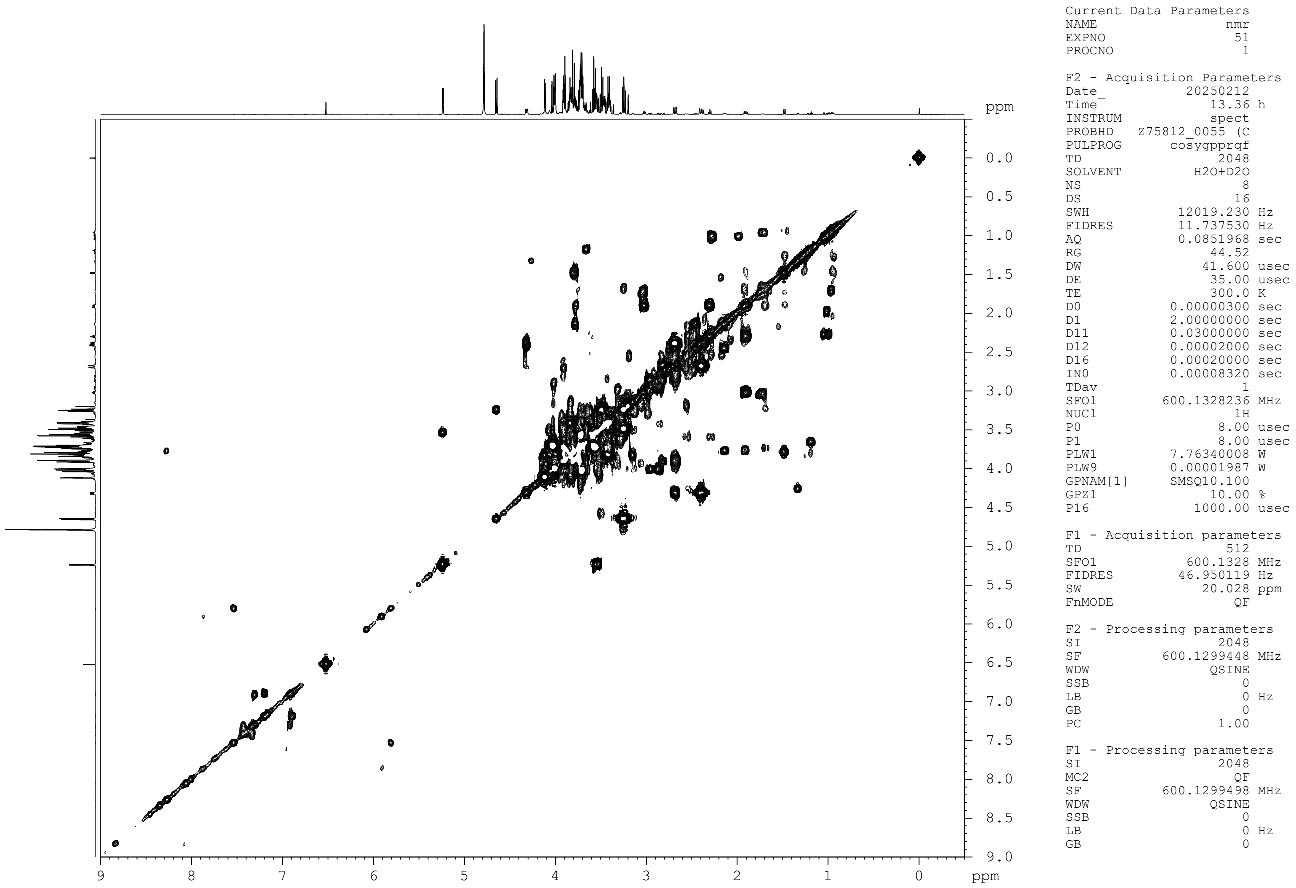

Figure S3: ^1H-^1H cosy spectrum of a representative zucchini juice sample.

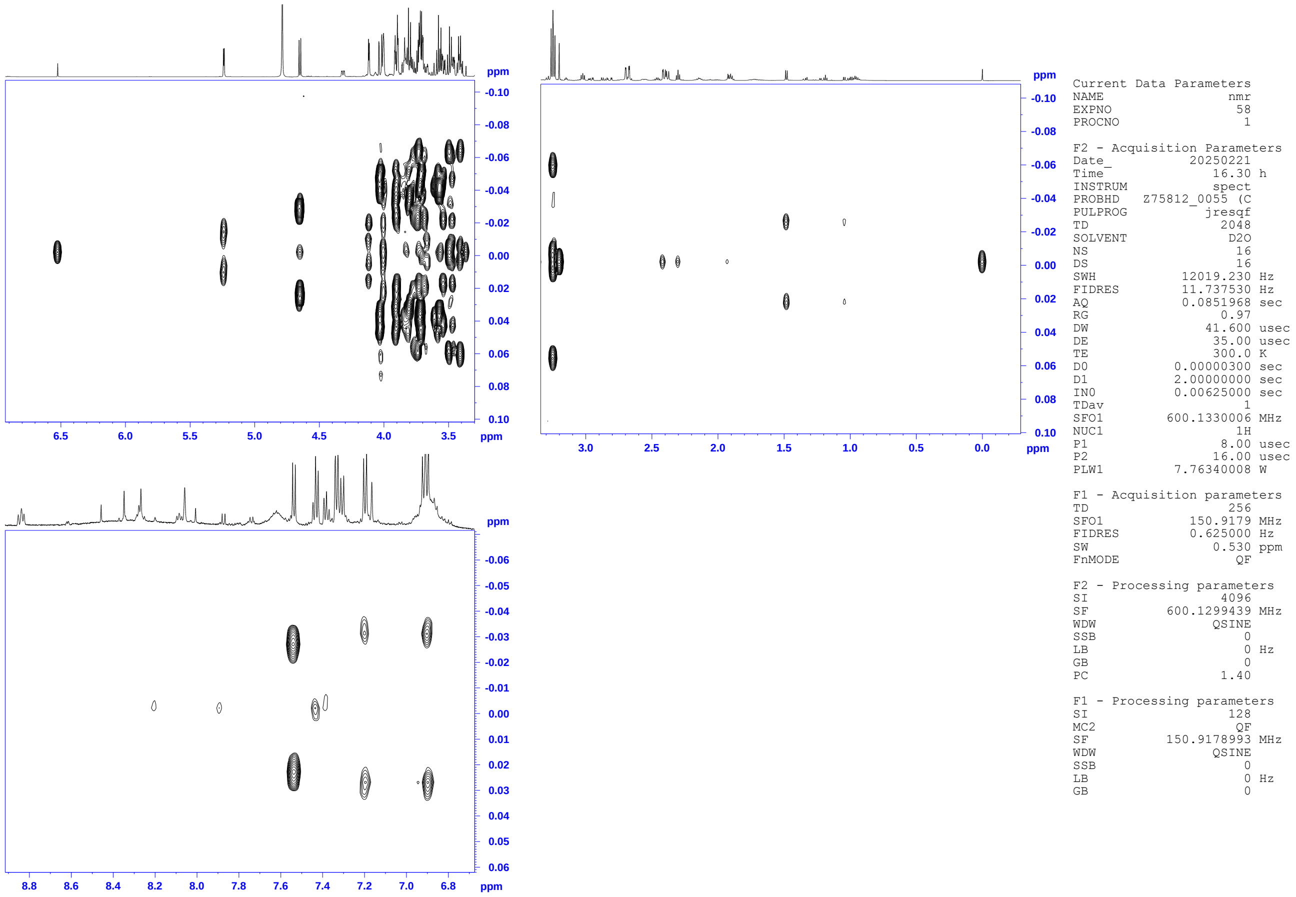

Figure S4: ^{1}H J resolved spectrum of a representative zucchini juice sample

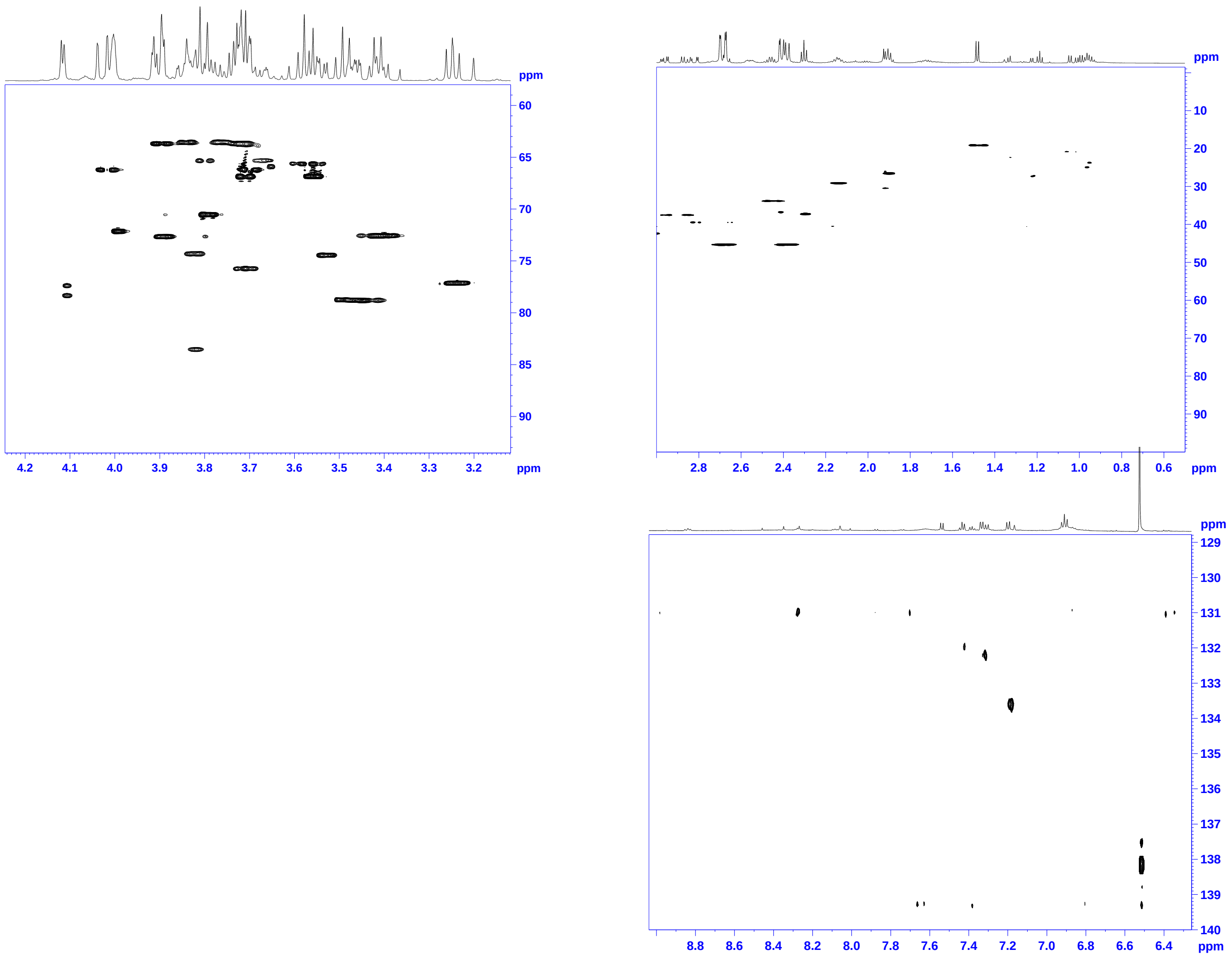

Figure S5: ^{1}H-^{13}C hsqc 2D spectrum of a representative zucchini juice samples. Expansions in the 0.5-3; 3-4.5; 6-9 spectral regions are represented.

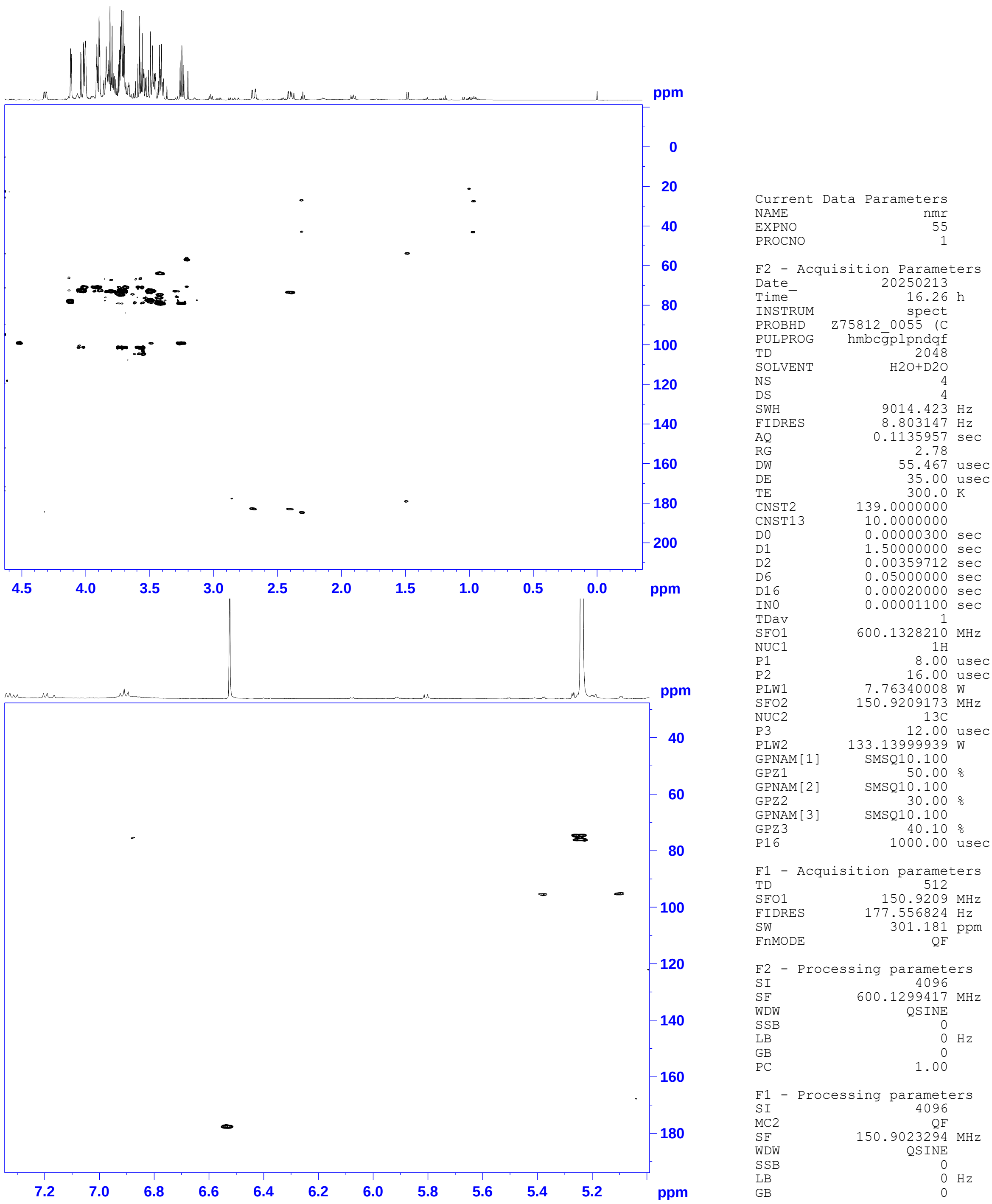

Figure S6: ^{1}H-^{13}C hmbc 2D spectrum of a representative zucchini juice sample. Expansions in the -0.5-4.5 and 5-7.5 spectral regions are represented.

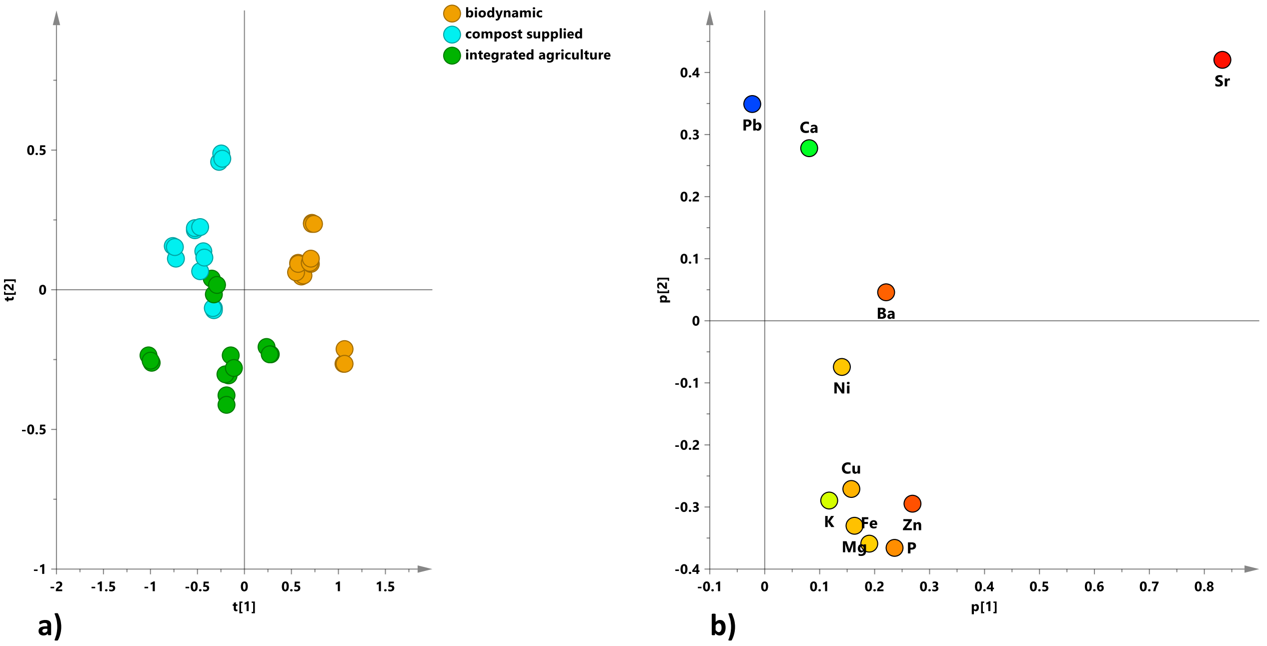

Figure S7 a) PCA score plot for the whole measured elements. b) loading scatter plot for the model

Table S1. List of biodynamic preparations and their effects. [Biodynamic Preparations Available online: https://demeter.net/biodynamics/biodynamic-preparations/]

Compost preparations	Effects
Yarrow preparation (P 502)	• promotes adaptability to the site<br>• has an invigorating effect<br>• regulates potassium metabolism in the plant<br>• regulates nitrogen, carbon, sulphur and potassium processes
Camomile preparation (P 503)	• makes the fertiliser more nitrogen-resistant
Nettle preparation (P 504)	• has a structure-improving effect on the soil
Oak bark preparation (P 505)	• promotes calcium processes<br>• inhibits fungal pathogens<br>• insecticide through tannic acid<br>• puts the shaping forces exactly where plant diseases might otherwise develop
Dandelion preparation (P 506)	• promotes potassium and silicic acid processes<br>• sensitises the plants to the environment<br>• strengthens the ability to attract nutrients
Valerian preparation (P 507)	• promotes phosphorus processes<br>• regulates heat processes in the soil, fertiliser and plant<br>• acts in flower and fruit formation
**Spray preparations**	**Effects**
Horn manure (500)	• stimulates soil activity<br>• promotes root growth<br>• activates the soil's own life<br>• supports soil loosening<br>• supports water and nutrient absorption<br>• promotes nitrogen fixation of the nodule bacteria
Horn silica (501)	• promotes and organises plant metabolism (photosynthesis)<br>• strengthens resistance to pests<br>• promotes harmonious growth and ripening processes<br>• ensures uniform ripening quality<br>• strengthens the aroma and improves storage capacity

Table S2. Technical data sheet of compost supplied "Bio Vegetal". Bio Vegetal is a biofertilizer with organic substance and 200 types of live microorganisms. [https://biovegetal.it/]

**Characteristics of organic substance**
presence of organic carbon<br>high content of humic and fulvic acids<br>balanced carbon-nitrogen ratio
**Triple effects of added microorganisms (Bacillus, Pseudomonas, Trichoderma, Mycorrhizae, etc.)**
Plant Growth Promoting (PGP)<br>induction of resistance to abiotic stress<br>induction of resistance to biotic stress
**Other benefits**
benefits for plants (improvement of the vegetative state, natural suppression of the main telluric phytopathogens, strengthening of natural defences to biotic and abiotic stress, reduction of the carbon footprint of agronomic production)<br>benefits for the soil (restoration of natural balance, improved water retention capacity, improvement of soil structure)<br>benefits for the environment (reduction of the water requirement of crops, reduction of CO_2 emissions, reduction of the use of mineral fertilizers)

Table S3. Concentrations of significant elements in zucchini samples expressed in ppm. Data were obtained by (ICP-AES) technique and expressed as means ± standard deviations. Results are indicated as the mean (± standard deviation) of different measurements for each of the three cultivation methods. Ca: calcium; P, phosphorus; Cu, copper; Fe, iron; Ni, nickel; Pb, lead; K, potassium; Mg, magnesium; Ba, barium; Sr, strontium; Zn, zinc.

Cultivations	P (ppm)	K (ppm)	Ca (ppm)	Mg (ppm)	Fe (ppm)	Zn (ppm)	Cu (ppm)	Ni (ppm)	Pb (ppm)	Ba (ppm)	Sr (ppm)
**BIODYNAMIC**	2724.23 ± 490.84	2481.06 ± 449.86	238.89 ± 52.95	113.54 ± 21.86	5.13 ± 2.06	2.60 ± 0.73	0.69 ± 0.08	0.12 ± 0.03	0.17 ± 0.04	0.20 ± 0.04	1.28 ± 0.24
**COMPOST SUPPLIED**	1135.42 ± 135.41	1795.23 ± 395.64	276.05 ± 86.87	67.89 ± 11.14	3.04 ± 0.73	1.14 ± 0.17	0.39 ± 0.05	0.08 ± 0.01	0.19 ± 0.02	0.12 ± 0.03	0.15 ± 0.06
**INTEGRATED AGRICULTURE**	1896.19 ± 349.35	2228.47 ± 538.62	201.58 ± 71.62	97.73 ± 23.98	4.05 ± 1.13	1.65 ± 0.50	0.57 ± 0.10	0.08 ± 0.01	0.12 ± 0.02	0.14 ± 0.05	0.17 ± 0.10
